# Unusual Manifestations of Vincristine Neuropathy: Report of Two Cases of Hodgkin Lymphoma

**DOI:** 10.4274/tjh.2013.0158

**Published:** 2014-09-05

**Authors:** Olga Meltem Akay, Eren Gündüz, Ercan Kaya, Mahmut Kebapcı, Zafer Gülbaş

**Affiliations:** 1 Osmangazi University Faculty of Medicine, Department of Hematology, Eskişehir, Turkey; 2 Osmangazi University Faculty of Medicine, Department of Otolaryngology, Head and Neck Surgery, Eskişehir, Turkey; 3 Osmangazi University Faculty of Medicine, Department of Radiology, Eskişehir, Turkey; 4 Anadolu Health Center, Bone Marrow Transplantation Center, Kocaeli, Turkey

**Keywords:** Vincristine, Neuropathy, Vocal cord paralysis, Paralytic ileus

## CASE 1

**Figure 1:**
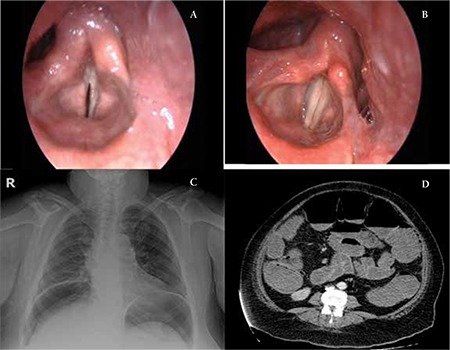
Videolaryngoscopy reveals immobile left vocal cord (A) and normal moving vocal cords (B) in case 1. Plain X-ray shows free air beneath the diaphragm (C) and abdominal CT shows dilated loops with intestinal pneumonitis (D) in case 2.

An 81-year-old man presented with a 6-month history of progressive cervical mass and weight loss. A biopsy specimen from a right supraclavicular lymph node was suggestive of Hodgkin disease of mixed cellularity/lymphocyte-depleted type. The clinical stage was IIB and the patient was started on combination chemotherapy with doxorubicin, bleomycin, vincristine (since vinblastine was not available on the market), and dacarbazine (ABVD). Two milligrams of vincristine was administered on days 1 and 14 of each 28-day cycle. After 2 courses (8 mg of vincristine), the patient complained of hoarseness. He noted weakness in his voice. Videolaryngoscopy revealed left vocal cord immobility with structurally normal-appearing cords (Figure 1A). His clinical profile and videolaryngoscopic findings were consistent with vinca alkaloid-induced vocal cord paralysis. Vincristine was omitted from the chemotherapy protocol and the patient’s hoarseness resolved gradually. Five months after stopping vincristine, the patient’s voice was much improved and his hoarseness disappeared. Repeat videolaryngoscopy showed normal vocal cords that moved freely (Figure 1B). Informed consent was obtained.

## CASE 2

A 43-year-old woman presented with a 3-month history of progressive dyspnea. Biopsy of a right supraclavicular lymph node showed classical-type mixed cellularity Hodgkin disease. The clinical stage was IIIA and the patient was started on combination chemotherapy with ABVD. Two milligrams of vincristine was administered on days 1 and 14 of each 28-day cycle. After 3 courses (12 mg of vincristine), the patient complained of mild abdominal pain and distension with constipation. Five days later, despite supportive care, her abdominal distension increased markedly and bowel sounds were not heard. An upright film of the chest revealed free air under the diaphragm (Figure 1C) and abdominal CT showed dilated loops with intestinal pneumonitis (Figure 1D). She underwent an emergent exploratory laparotomy. Since she had a perforation in the cecum measuring approximately 2 cm as well as small and large bowel dilatation, right hemicolectomy/ileotransverstomy was performed. A diagnosis of paralytic ileus due to autonomic neuropathy induced by vincristine was made. Due to severe sepsis, the patient rapidly deteriorated postoperatively and died 2 weeks after the operation.

Vocal cord paralysis is an unusual manifestation of vincristine neurotoxicity. Most cases present with unilateral nerve palsy, and hoarseness of voice is the most common presenting symptom [1,2,3,4]. Vinca alkaloid-induced myenteric nerve damage may contribute to paralytic ileal and cecal distension that further enhances intestinal ischemia [5]. Since these complications are not always realized, prompt recognition is imperative for avoiding severe dysfunction of several organ systems. Informed consent was obtained.

## CLINICAL PICTURES IN HEMATOLOGY

A 59-year-old Middle Eastern man with no significant past medical history was referred to the hematology clinic of our hospital for incidentally detected anemia on routine blood examination. When seen, he complained of chronic mild fatigue for 1 year. The rest of the review of systems was unremarkable. He did not take any medications and did not smoke or drink alcohol. His diet was significant for his daily intake of liver. Physical exam showed normal vital signs and was otherwise unremarkable except for pallor.

A complete blood count revealed hemoglobin of 73 g/L, total RBC count of 4.41x1012/L, MCV of 54.2 fL (low), MCH of 16.5 pg (low), MCHC of 304 g/L, RDW of 29.9%, and platelet count of 262x109/L. The reticulocyte count was 2.3% with a low reticulocyte production index, suggesting a hypoproliferative anemia. Serum ferritin level was 1336 ng/mL (normal: 24-336 ng/mL), serum vitamin B12 level was 302 pg/mL (normal: 180-914 pg/mL), and folic acid level was 4.5 ng/mL (normal: 3.5-16.1 ng/mL). A hemoglobin electrophoresis test was normal and the sickle test was negative. Erythrocyte sedimentation rate was 2 mm/h. A bone marrow aspiration/biopsy was performed, which showed a hypercellular marrow with trilineage hematopoiesis with maturation. There was microcytic anemia with erythroid hyperplasia, increased stainable iron, and 60% ringed sideroblasts, consistent with sideroblastic anemia (Figures 1, 2). There was adequate granulopoiesis and megakaryopoiesis. The flow cytometry of the bone marrow sample was negative. Fluorescent in situ hybridization was negative for chromosomal abnormalities and cytogenetic analysis showed a normal male karyotype. Based on the above findings, a working diagnosis of myelodysplastic syndrome (MDS; refractory anemia with ringed sideroblasts) was made. It was decided to observe his counts on a regular basis. Informed consent was obtained.

In the meantime, the patient started taking over-the-counter micronutrient and mineral supplements that contained copper and zinc amongst others. At the 6-month follow-up when a complete blood count was repeated, it showed a much-improved hemoglobin level of 127 g/L with MCV of 80.4 fL and normal MCH of 26.3 pg, raising the possibility of a reversible process causing sideroblastic anemia. Since the counts had corrected to near normal, he was observed. On the subsequent visit 6 months later, the patient was seen in the clinic, and he had not been taking his micronutrient and minerals pills for 3 weeks. His hemoglobin fell to 107 g/L and MCV to 66.3 fL. At that time, a test of copper and zinc levels was done, which revealed low zinc levels of 129.99 µmol/L (normal: 154.75-495.2 µmol/L) and a normal copper level of 1.4 µmol/L (normal: 1.3-2.89 µmol/L). The mineral pill was restarted, and his hemoglobin improved to 117 g/L and MCV to 73.8 fL after 2 months. He continues to be asymptomatic with stable blood counts.
